# A 3D Printed Mini-Gel Electrophoresis System for Rapid and Inexpensive DNA Nanoswitch Biosensing

**DOI:** 10.1021/acsmeasuresciau.6c00041

**Published:** 2026-05-21

**Authors:** Vinod Morya, Andrew Hayden, Lifeng Zhou, Dadrian Cole, Ken Halvorsen

**Affiliations:** † The RNA Institute, University at Albany, State University of New York,Albany,New York 12222, United States; ‡ Southern University of Science and Technology,Shenzhen 518055,China

**Keywords:** gel electrophoresis, separation science, DNA nanoswitches, DNA nanotechnology, biosensing, 3D printing

## Abstract

Gel electrophoresis has been a cornerstone laboratory technique for decades, yet it is often viewed as cumbersome and costly and confined to laboratory settings. Recent advances in DNA nanotechnology have used electrophoresis as a primary readout for some biosensing applications such as DNA nanoswitches, where a conformational change in a DNA structure indicates the presence of a target molecule. Conventional gel electrophoresis setups are not ideal for such targeted applications, with moderate equipment cost, excessive reagent use, and time-consuming processes. Here, we adopt a reductionist, application-driven approach to redesign gel electrophoresis specifically for DNA-nanoswitch-based detection. We present a fully 3D printable mini-gel electrophoresis system that incorporates conductive plastic electrodes, demonstrating performance comparable to conventional systems using platinum electrodes. By optimizing the interelectrode distance and running parameters, our system resolves the on/off states of DNA nanoswitches in as little as 1 min. We further show that the device operates reliably at low voltages, including when powered by a USB power bank, and even enables instrument-free nanoswitch readout using an LED with a cell phone camera. Our design substantially reduces the cost, voltage requirements, material usage, operational complexity, and experimental time. These improvements make gel-based biosensing more practical outside traditional laboratory environments, paving the way for the broader adoption of gel electrophoresis in point-of-care and resource-limited settings.

## Graphical abstract

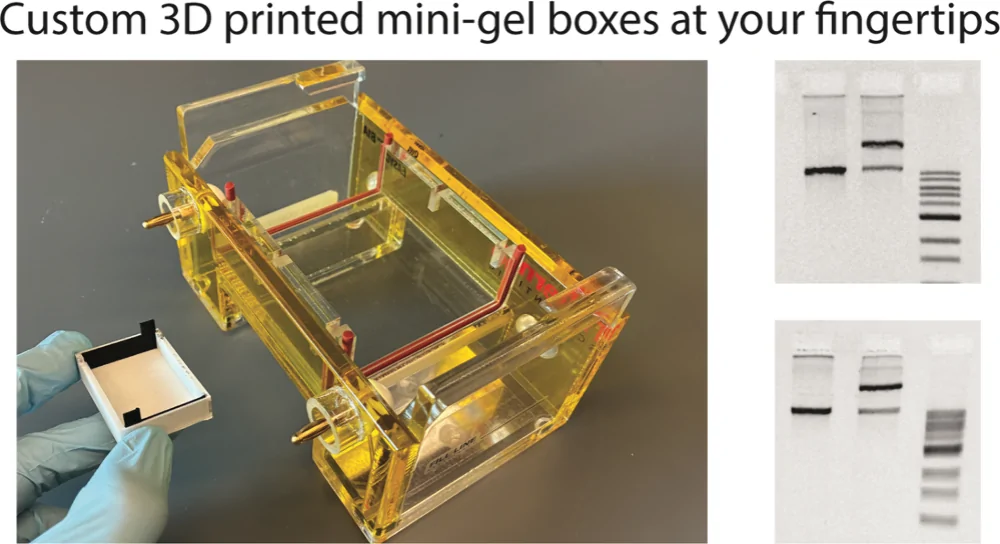

## Introduction

Gel electrophoresis is a common and foundational laboratory technique for separating charged polymers by size, topology, or conformation. Perhaps the most widely used format is agarose gel electrophoresis of DNA and RNA, which has remained largely unchanged since its introduction in 1972.^1^ The method is deeply integrated into molecular biology workflows, including cloning, PCR analysis, genetic identification, and nucleic acid quality assessment.^2,3^ Despite its ubiquity, the typical equipment has seen minimal evolution. Standard laboratory gel boxes rely on large hand-cast slab gels (often ∼50–200 cm^2^), paired with sizable buffer reservoirs (∼0.4–2 L), and require relatively high voltages (50–150 V) delivered by benchtop power supplies.^4^ Visualization of the separated nucleic acids typically relies on intercalating dyes and costly gel documentation systems, which adds expense and operational constraints. Together, these elements make traditional gel electrophoresis confined to laboratory settings, moderately expensive to run, and a source of substantial chemical waste. Remarkably, commercial gel boxes are still strikingly similar to designs introduced in the 1970s, more than half a century ago.^5,6^

Still, electrophoresis has experienced some innovation, including advancements in materials, miniaturization, microfluidic electrophoresis, and capillary electrophoresis systems.^7–10^ However, these innovations have largely emerged to address analytical performance at the research or clinical scale, rather than to rethink the basic accessibility, size, or operability of the standard slab-gel format. Little effort has focused on redesigning agarose electrophoresis for simplicity, portability, or low-resource settings. In recent years, electrophoresis has also taken on a new role as a primary readout for some biosensing assays.^11–13^ Rather than merely separating nucleic acids, electrophoresis can directly report on molecular detection events through mobility shifts. This shift in usage opens the possibility that electrophoresis could support low-cost diagnostic platforms, if the technique itself can be simplified and adapted for point-of-care (POC) contexts.

We have previously developed DNA nanoswitches that use gel electrophoresis as a readout for detecting a wide range of biomolecules.^14–18^ DNA nanoswitches operate by converting a molecular recognition event into a topological change in the DNA structure (Figure 1a).^19^ When the nanoswitch binds its target, it undergoes a conformational change, shifting from a linear to a looped state, producing a clear mobility shift during electrophoresis (Figure 1b). Importantly, the platform is fully programmable and has been adapted to detect diverse classes of biomarkers, including DNA,^19^ various RNA species,^14,15^ proteins,^20^ and combinations of molecular targets.^21^ The nanoswitch system is highly cost-effective (typically <1 cent per nanoswitch), versatile, and sensitive. Despite these advantages, reliance on traditional gel electrophoresis has been one of the main criticisms of nanoswitch-based detection, particularly for use in POC diagnostics or resource-limited environments.

**Figure 1:**
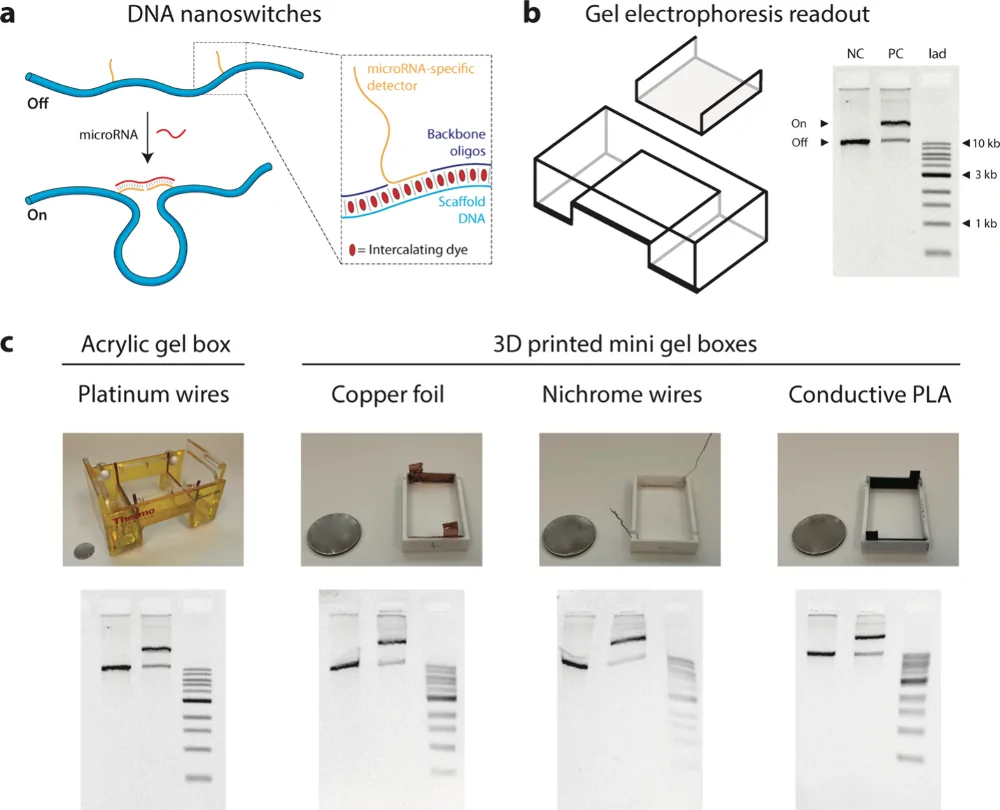
DNA nanoswitch concept and development of custom gel boxes. (a) DNA nanoswitches are designed to reconfigure from linear to looped in the presence of a molecular target. (b) Agarose gel electrophoresis enables identification of the linear (unlooped) and looped forms. (c) Comparison of a standard gel setup with custom-made 3D printed mini-gel boxes using copper, nichrome, or conductive PLA electrodes. Lanes in all the gels from left to right: 1 = NC (negative control, no target in the reaction), 2 = PC (positive control, 1 nM target in the reaction), and 3 = 1kb DNA ladder. All gels were run at 5.5 V/cm for 45 min (metal disc, the size and shape of a US quarter for scale).

Here, we set out to streamline and reengineer gel electrophoresis as a dedicated readout for DNA nanoswitches. The mobility shift between the on- and off-states is large enough that full-scale electrophoresis is unnecessary. We first miniaturized the gel system to allow lower voltage operation, decrease the running time, and reduce the cost of both manufacturing and operation. We evaluated various electrode materials and found the best performance and cost-effectiveness using a fully 3D printed design with conductive plastic electrodes. With this system, we demonstrate nanoswitch detection in just a few minutes at voltages compatible with standard battery or USB power bank operation. Finally, we evaluated dyes, lighting strategies, and low-cost optical filters to enable imaging with a cell phone camera or even visible detection by eye without the need for specialized instruments. We anticipate that these efforts represent an early step toward a new generation of gel-based POC assays for health care, environmental monitoring, and low-resource diagnostics.

## Results

Our primary goal in this work was to reimagine gel electrophoresis as a readout for DNA nanoswitches—particularly to optimize the system to reduce cost, time, voltage, and experimental effort. We took a reductionist approach to determine the minimal requirements, and the first consideration was that resolving our DNA nanoswitches requires only a small fraction of the area of a typical gel. The smallest commercial mini-gel system we use (Owl B1A) still exceeds the practical requirements of our application, with a gel size of 7 × 8 cm, a footprint of about 15 × 10 cm, and holding ∼400 mL of running buffer. In contrast, our DNA nanoswitch bands occupy only ∼0.5 × 0.5 cm (less than 1% of the total gel area) for a single lane. We considered that a smaller gel footprint could offer many potential advantages including closer electrodes for lower voltage operation, less reagent use including agarose and running buffer, and lower overall equipment cost. While there is a potential loss in resolving power in using a shorter gel, this is not expected to be a problem when resolving two well-separated bands produced by DNA nanoswitches. To evaluate a smaller gel design, we 3D printed a plastic (PLA) tray and comb to accommodate 3 gel lanes in a 40 mm long gel box (Figure 1c). Based on our previous use of bufferless gel electrophoresis (E-gel system, Thermo Fisher), we hypothesized that a large buffer reservoir was unnecessary for our application and that a few mL of buffer to overlay the gel would suffice.

Most standard gel electrophoresis systems use platinum wires, which are known for high conductivity and low corrosion but are also extremely high cost. For our mini-gel box, we considered three lower-cost materials to use as electrodes—copper foil, nichrome wire, and conductive plastic (Figure 1c). We found that all three materials worked, and we were pleasantly surprised to see that conductive PLA had the straightest bands and best resolution among the three. The conductive PLA is also the easiest to work with since it can be entirely 3D printed and does not exhibit visible surface fouling due to oxidation as the copper and nickel wires did. Overall, we found the performance of our 3D printed mini-gel with conductive plastic electrodes and no buffer reservoirs performed similarly to our conventional gel system with platinum electrodes that we used as our gold standard benchmark.

Considering the good performance and versatility of our 3D mini-gel design, we decided to move forward with this design for further optimization. First, we tested whether a buffer gap at each electrode was beneficial by running identical gels with or without the gap (Figure S1). We found that both systems produced suitable results, but we noticed that a small gap helped reduce the entrapment of bubbles generated at the electrodes, potentially helping to reduce the pH stress and allow better heat dissipation. On the basis of this, we decided to include a 1 mm gap between the gel and each electrode for future studies. Next, we assessed repeatability by printing 5 identical gel boxes and running fresh gels in each one (Figure S2). We also assessed reuse of the same gel box multiple times by running 5 sequential gels in one box. We found that the performance degraded slightly over time but still produced acceptable results after 5 runs. We also compared the effectiveness of 3D printed combs with an acrylic comb (Figure S3). We found that the 3D printed combs produced indistinguishable results from the acrylic comb, and all print qualities also gave similar results.

A primary advantage of 3D printing is its rapid and inexpensive prototyping. We took advantage of this capability to evaluate the effect of interelectrode distance on our 3D printed mini-gel, which is not a parameter that can be changed easily in standard lab electrophoresis. Since the driving force for electrophoresis comes from the electric field strength (i.e., nominally the voltage divided by the interelectrode distance), moving the electrodes closer should allow for separation at lower total voltages. We designed and 3D printed gel boxes with interelectrode distances ranging from 10 to 40 mm and ran gels, keeping a constant nominal electric field (Figure 2). Migration in the largest boxes of 30–40 mm was very similar to the owl reference gel, while the smaller gel boxes started to exhibit some band compression (Figure 2b,d). In principle, a constant electric field would produce uniform migration across all gel boxes, so these results suggest some confounding factors that are likely changing field strength. For our DNA nanoswitch studies, we identified the 15 and 20 mm spacings to be the best mix of low voltage and a box and gel still large enough to physically handle without much trouble.

**Figure 2:**
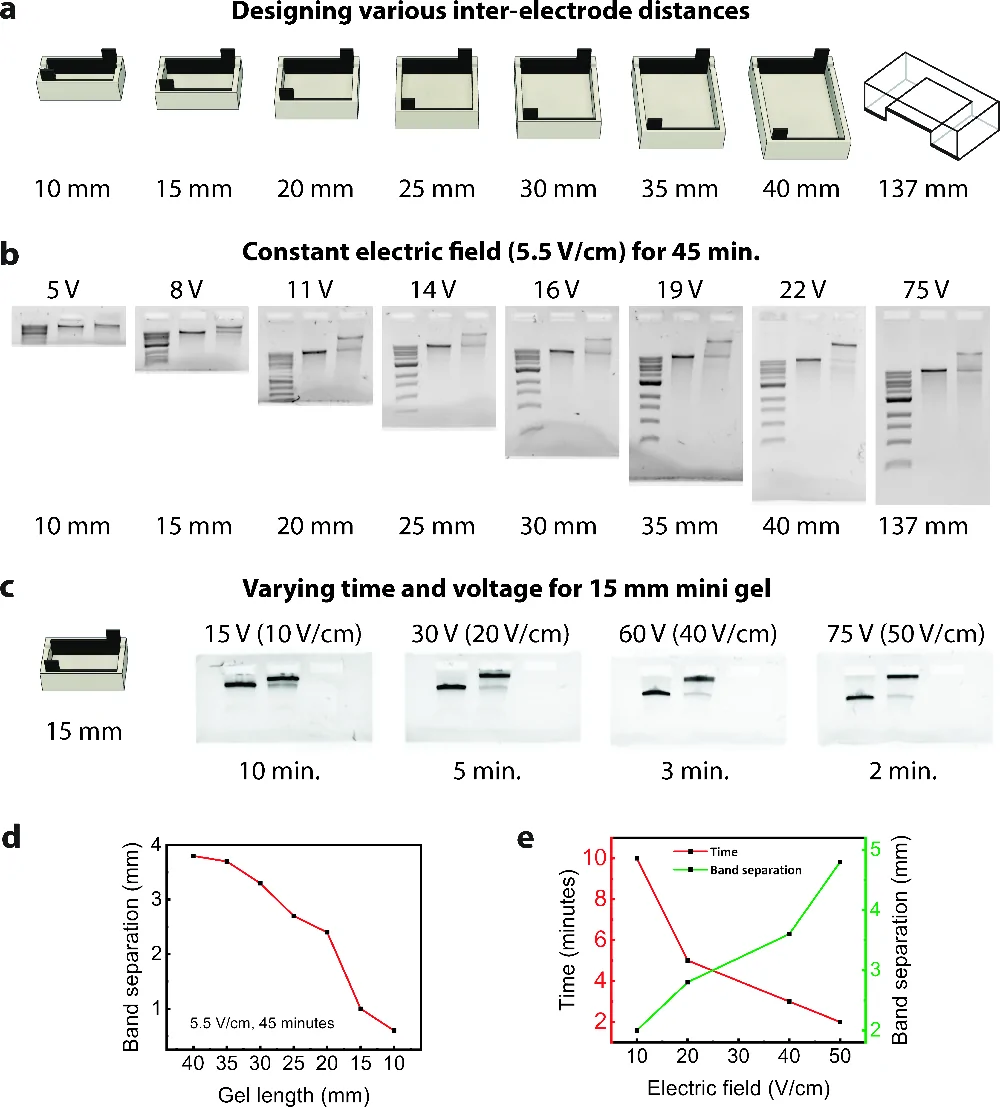
Optimization of electrode spacing. (a) Cartoon showing how reducing interelectrode length and voltage can maintain the same electric field and DNA mobility. (b) Design and testing of mini-gel boxes with various interelectrode distances and comparisons against a commercially available gel box. Lanes in all the gels from left to right: 1 = 1kb DNA ladder, 2 = negative control, 3 = positive control. (c) Testing different times and voltages for the 15 mm mini-gel box. Lanes in all the gels from left to right: 1 = negative control, 2 = positive controls, 3 = empty. (d) Relation between band resolution and interelectrode distance, as analyzed from panel (b). (e) Optimization of electric field and run time for rapid resolution of DNA nanoswitch states in 15 mm gel boxes, as analyzed from panel (c).

Proceeding with the 15 mm design, we further optimized the running conditions with the goal of achieving separation in a few minutes. We first tested different gel percentages with constant running conditions and found relatively similar results across percentages of 0.7 and 1.2%. We chose 0.8% for further experiments, as it gives a better balance between band resolution and agarose used (Figure S4). We next systematically increased the applied voltage while proportionally decreasing the run time to evaluate the minimum time required to achieve a band resolution. (Figure 2c). An increased electric field enables faster and improved nanoswitch resolution. We found that we could run the gel with electric field strengths as high as 50 V/cm, allowing the DNA nanoswitches to be resolved in as little as 2 min (Figure 2e). It is interesting to note that this field strength would correspond to >650 V in our Owl mini box, outside of the recommended operating range of the Owl box and of many voltage supplies.

Moving toward use outside of a lab environment, we considered which voltages would be easily accessible by battery power for nonlab use. We tested a 1.5 V AAA battery (4 in series, 6 V), 1.5 V coin cell (4 in series, 6 V), and a 9 V battery. We were able to resolve our nanoswitches using all of these power sources, with varying time requirements (Figure 3a,b). The use of portable batteries eliminates the requirement for costly standard benchtop power supply systems. To further remove the need for expensive and bulky gel imaging systems, we demonstrate that SYBR Safe staining, in combination with a blue-light source and an orange safety filter, enables gel detection using a standard cell phone camera (Figure 3c,d). To bring all of these innovations together, we demonstrate end-to-end detection of human rRNA from total RNA extracted from HeLa cells (Figure 3e). We tailored our nanoswitch to detect 5S rRNA, a ∼120 nt subunit of the eukaryotic ribosome, which we have previously tested.^18^ A synthetic DNA mimic of 5S rRNA was used as a positive control, and the total RNA sample (250 ng/μL) isolated from HeLa cells was used as a test sample. We found that using our mini-gel, we were able to successfully detect the 5S rRNA with results comparable to the gel run on a traditional Owl box (Figure S5).

**Figure 3:**
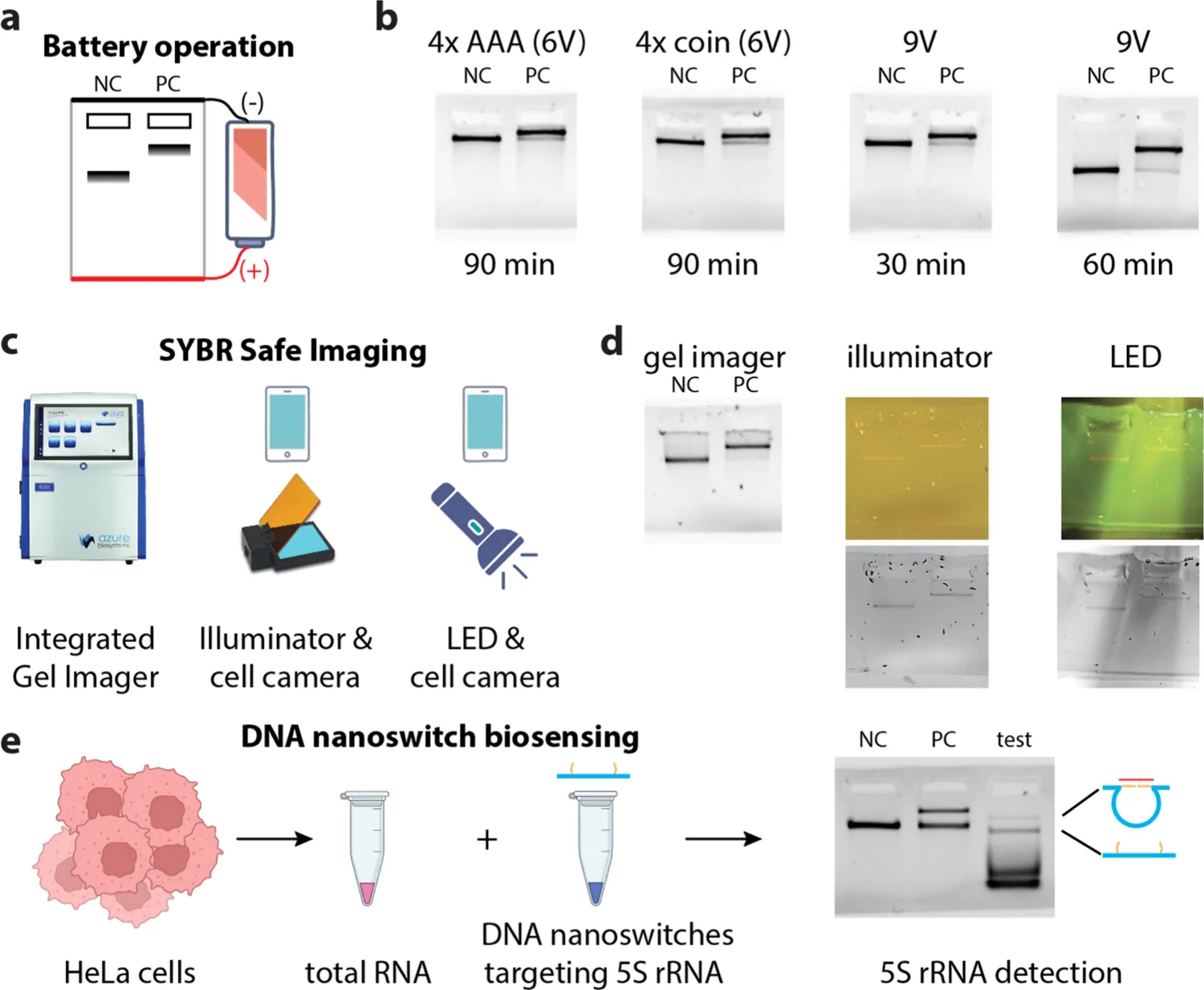
Instrument-free operation and imaging and analyzing a total RNA sample. (a) Mini gels allow low voltage operation compatible with battery power; (b) three different power sources were tested, with gel results at various times; (c) cartoon of required components for gel imaging; (d) a single gel imaged with three variants of gel imaging; (e) detection of 5S rRNA in a total RNA sample isolated from HeLa cells in a 15 mm mini-gel box.

## Discussion

Here, we have shown that gel electrophoresis systems can be redesigned and rapidly and inexpensively manufactured to suit the needs of specific applications such as biosensing. For our DNA nanoswitches, we demonstrate that fully 3D printed mini-gel boxes can save money, time, and materials for identifying biomolecules when compared with commercially available gel systems. For the nanoswitch assay in particular, we have shown that we can get the same result with ∼1000× lower equipment cost, ∼100× lower buffer volume, ∼10× lower voltage, ∼10× lower reagent cost, and ∼10× lower time. Our solution is transformative enough to potentially turn thousands of dollars of equipment into low-cost consumables.

Agarose gel electrophoresis has not changed much, but there have been some progressions of techniques such as the advent of capillary gel electrophoresis,^22^ Microplate Array Diagonal-Gel Electrophoresis (MADGE),^23^ and pulsed field electrophoresis.^24^ Commercially, several all-in-one gel electrophoresis systems have been developed to simplify operation, such as E-Gel,^25^ BlueGel,^26^ and MiniOne.^27^ While E-Gel is primarily designed for research applications, systems like BlueGel and MiniOne are largely geared toward educational use. In parallel, advances in nucleic acid stains, such as GelRed, have improved safety by reducing toxicity while enhancing fluorescence sensitivity. While use of gel electrophoresis outside of a lab environment is uncommon due to general need for high voltages, large buffer volumes, and gel preparation, the cartridge-based E-gel system provides a notable exception. It uses “buffer less” gels and can be paired with a power bank and reader to perform and analyze electrophoresis. We took some inspiration from this system to reimagine gel electrophoresis for our specific application of DNA nanoswitch biosensing.

Most gel electrophoresis systems tend to be “one-size-fits-all”, which can be useful for a lab environment but can also be wasteful and inefficient for targeted applications such as our DNA nanoswitches. Some other common uses of electrophoresis also only use a small subset of gel area, including validating PCR products,^3,28^ plasmid insertion,^29^ and DNA origami.^30^ For these types of applications, the typical large-format gel has many drawbacks including higher cost of equipment and reagents, higher running voltage and power consumption, and longer preparation times. Most of these drawbacks are driven by a single design choice – distance between electrodes. Closer electrode spacing reduces overall footprint/cost, volume, running voltages, and overall power. Reducing gel dimensions does come with a fundamental trade-off, as a shorter separation distance can limit resolution under standard electrophoretic conditions. Overcoming this requires reoptimization of operating parameters, particularly electric field strength and run time, to preserve sufficient separation between species despite reduced gel length.

At the heart of our innovations here is the enabling technology of 3D printing for prototyping and manufacturing. 3D printing enables affordable, rapid, on-site mass production in virtually any setting, and has become increasingly common in research.^31–33^ What was once a specialized and complex process in the early 2000s has become consumer-facing and user-friendly. It is worth pointing out that this work was 3D printed on a consumer-level printer first introduced in 2016 and hardly state-of-the-art after 10 years. Use of 3D printing has been slower to catch on in scientific research, but use is increasing. We have previously used 3D printing in our lab for the development of the Centrifuge Force Microscope,^34,35^ and others have used it to build light microscopes,^36^ centrifuges,^37^ microfluidics,^38^ rehabilitation devices,^39^ and other small laboratory equipment.^40^ For this gel project, the printable electrodes were key to enabling rapid and inexpensive printing and testing of various designs and electrode spacings. We include our design files for others to use (Supplemental File 1).

Aside from performance considerations, the mini-gel system has a large cost advantage over commercial systems. Lab-grade gel electrophoresis equipment can cost up to tens of thousands of dollars for a gel box, a high-voltage power supply, and imaging equipment. Our 3D printed custom gel box was produced for ∼$0.30 (and could likely be optimized to reduce cost below $0.10), and the assay can be run on battery power outside of a lab environment. For reference, a standard Thermo Scientific Owl EasyCast gel box costs >$1000 (excluding imaging), and power supply each costing over $500. These costs and electrical requirements of conventional gel electrophoresis equipment have prevented agarose gels from being realized as an option for use outside the lab environment.

This work narrows the gap to achieving POC utility with DNA nanoswitches and a gel-based readout. Several applications that have already been developed such as viral RNA detection may benefit from such a format, potentially enabling healthcare providers to conduct diagnostic tests and monitor patients’ health status in real time, at or near the POC. This would be particularly useful in remote and resource-limited settings where access to conventional laboratory facilities is limited. The 3D printed gel boxes can be used as disposables or can be reused multiple times, and it may be possible for gels to be precast and vacuum-sealed for immediate use at the POC. Bands can be seen with as little as a blue flashlight and a cell phone camera. Further improvements in bulk manufacture, storage, transport, and user guidelines would still be needed, but we believe that we have demonstrated that a gel readout can be a viable option.

Beyond POC, we hope that our developments here will encourage other researchers to think “outside of the box” when considering electrophoresis. Just because it has been a one-size-fits-all lab technique that is tried and true does not mean it cannot be improved or made more efficient. There is still substantial room for innovation, and this work just scratches the surface. Customization of gel box size and shape, comb shapes and number of wells, and electrode position and orientation are all possible. These customizations may provide additional utility for various applications without substantial additional cost.

With inexpensive reconfiguration to suit individual researchers’ needs, we see applications that certainly could go far beyond our own DNA nanoswitch sensor. We hope researchers might consider, the next time they prepare liters of buffer and run gels in equipment costing thousands of dollars, whether such setups are truly ideal for their specific application or simply a matter of convention. This work demonstrates that thoughtful engineering and purposeful simplification can extend gel electrophoresis beyond traditional laboratory constraints and open new possibilities for accessible, application-driven use.

## Experimental Section

### Mini-Gel Design and Fabrication

All mini-gel models were designed using Autodesk Fusion 360 (version 2605.1.39). Models were exported in “.stl” format into Ultimaker Cura (v. 5.9.1) 3D printing software for formatting. G-code files were exported and printed on a dual-extruder Ultimaker 3 FFF 3D printer (PN:9671) equipped with two Ultimaker AA 0.4 mm print cores (PN:9529). All mini-gel box models, unless otherwise specified, were prepared using default normal resolution (0.15 mm) print settings for 2.85 mm Ultimaker White or Black Tough PLA (Poly Lactic Acid) with 100% infill, lines infill settings. Mini-gel electrodes were prepared using 2.85 mm ProtoPasta Electrically Conductive Composite PLA with these settings: nozzle temperature 230 °C, 100% infill, and lines infill settings. All mini-gel systems were used without further post processing.

Portable power was supplied using alkaline batteries, including four Procell AAA 1.5 V cells, four Energizer LR44/A76 1.5 V button cells, and a Procell 9 V alkaline battery. These configurations were used to provide nominal output voltages of 6 and 9 V for device operation. The battery power source was directly connected to the anode and cathode terminal posts of the mini-gel by using a pair of alligator clips with jumper wires.

### DNA Nanoswitch Reactions

All optimization experiments were performed using a previously reported DNA nanoswitch designed to detect Let-7b miRNA. The nanoswitch reactions, unless otherwise specified, were performed at 30 °C in 20 mM Tris-HCl (pH 8) supplemented with 30 mM magnesium chloride (MgCl_2_) with equimolar amounts of Let-7b nanoswitch and analyte (DNA mimic of Let-7b miRNA, 5′-TGAGGTAGTAGGTTGTGTGGTT-3′) with a reaction concentration of ∼400pM. Nanoswitch reaction mixtures were incubated for 1 h, then 2:1 6× loading dye: 10× DNA stain (Biotium GelRed) was added to a final concentration of 1× and mixed. Ten microliters of sample were loaded to each well and electrophoresed at various voltage–times as specified in the “Results” section. Total RNA was extracted from HeLa cells (BioChain, Cat# R1255811–50) and diluted to a final concentration of 250 ng/μL prior to use as input material for target detection with the DNA nanoswitch. All the buffers and reagents were prepared using nuclease-free water (Cytiva, Cat# SH30538).

### Agarose Gel Electrophoresis

All agarose gels used in all the experiments were prepared from Sigma-Aldrich agarose, Bioreagent low EEO (Cat. No. A9539), and freshly prepared 0.2 μm filter-sterilized 0.5X tris-borate-EDTA buffer (TBE), unless otherwise specified, in a 0.8% w/v ratio. A 1kb DNA ladder from New England Biolabs (Cat# N3232) was used as a reference sample. Standard agarose gels contained 25 mL of agarose gel and were prepared and run on a Thermo Scientific Owl EasyCast B1A Mini Gel electrophoresis system (Cat# B1A-BP). Mini-gels were cast in 3 mm thick slabs using variable volumes of agarose gel depending on the following volumetric ratio: 750 μL molten agarose per 10 mm gel length (Supporting Information, Table S1). All AGE, except battery-powered mini-gels, were run in 0.5X TBE running buffer at constant voltage using a Labnet International Inc. Enduro 300 V power supply (Model: E0303) with the voltage–times specified in the “Results” section.

### Gel Imaging

All gel images for optimization experiments were captured by an Azure BioSystems Azure 400 Bioanalytical Imaging System. Gels were imaged with ethidium bromide or SYBR Safe settings. Various exposures (500 ms to 20 s) were taken to maximize sensitivity while minimizing pixel saturation. Raw TIFF files were exported to Bio-Rad Image Lab software (ver. 6.1) for analysis. To capture the gel images by a cell phone, the gel stained with SYBR Safe was illuminated by a blue LED (Thorlabs LED465E), and an orange safety filter was used.

## Supplementary Material





## Notes

Figure S1: effect of electrode-gel spacing on gel performance; Figure S2: repeatability of mini gels; Figure S3: use of acrylic vs 3D printed gel combs; Figure S4: use of various gel percentages in resolving DNA nanoswitches; and Figure S5: target detection in a total RNA sample with traditional gel box vs mini-gel box; Table S1: volume of agarose gel and running buffer used for different gel length (PDF)
40mm box+comb+spacer files in both 3MF and STL formats (ZIP)
