# Supplementary material for: A 3D Printed Mini-Gel Electrophoresis System for Rapid and Inexpensive DNA Nanoswitch Biosensing
Source: ACS Meas Sci Au. 2026 May 21;6(4):1049–55. doi: 10.1021/acsmeasuresciau.6c00041 (PMC13495711; doi:10.1021/acsmeasuresciau.6c00041)
Supplement: Supplementary file 1 [file tg6c00041_si_001.pdf]

Supplementary Information:

A 3D printed mini-gel electrophoresis system for rapid and inexpensive DNA nanoswitch biosensing

Vinod Morya[1], Andrew Hayden[1], Lifeng Zhou[1,2], Dadrian Cole[1], Ken Halvorsen[1]*

[1]The RNA Institute, University at Albany, State University of New York, Albany, NY, 12222 USA.

[2]current address: Southern University of Science and Technology, Shenzhen 518055, China

*Correspondence: khalvorsen@albany.edu

Table of Contents:

Table S1: Volume of agarose gel and running buffer used for different gel length.

Figure S1: Effect of electrode-gel spacing on gel performance

Figure S2: Repeatability of mini gels

Figure S3: Use of acrylic vs 3D printed gel combs

Figure S4: Use of various gel percentages in resolving DNA nanoswitches.

Figure S5: Target detection in a total RNA sample with traditional gel box vs mini-gel box.

Table S1. Volume of agarose gel and running buffer used for different gel length.

Gel length (mm)	Agarose gel volume (mL)	Running buffer (mL)
10	0.75	0.75
15	1.1	1.1
20	1.5	1.5
25	1.8	1.8
30	2.2	2.2
35	2.6	2.6
40	3	3

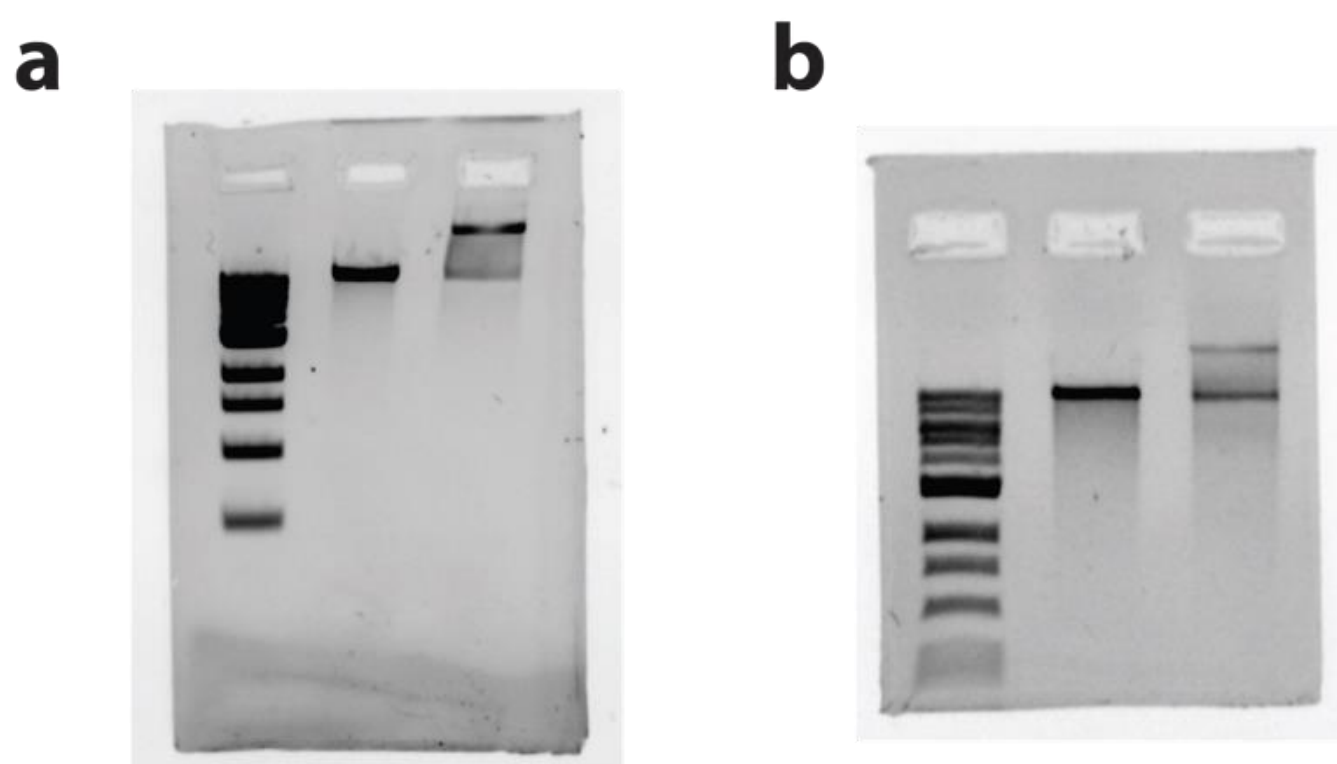

Figure S1. Effect of electrode-gel spacing on gel performance. a) Agarose gel run in a 40 mm Minigel box with no gap between the electrodes and the gel. b) Agarose gel run with a 3 mm gap between the electrodes and the gel on each side. While both configurations enable electrophoretic separation, some visible differences are observed. The gel run without gaps exhibits slower migration and visible distortion of the gel matrix near the bottom edge, likely due to localized heating and partial melting. In contrast, the gel run with electrode-gel gaps shows slightly higher migration and no observable gel deformation. Lanes from left to right: 1= 1kb DNA ladder, 2= negative control, 3=positive control.

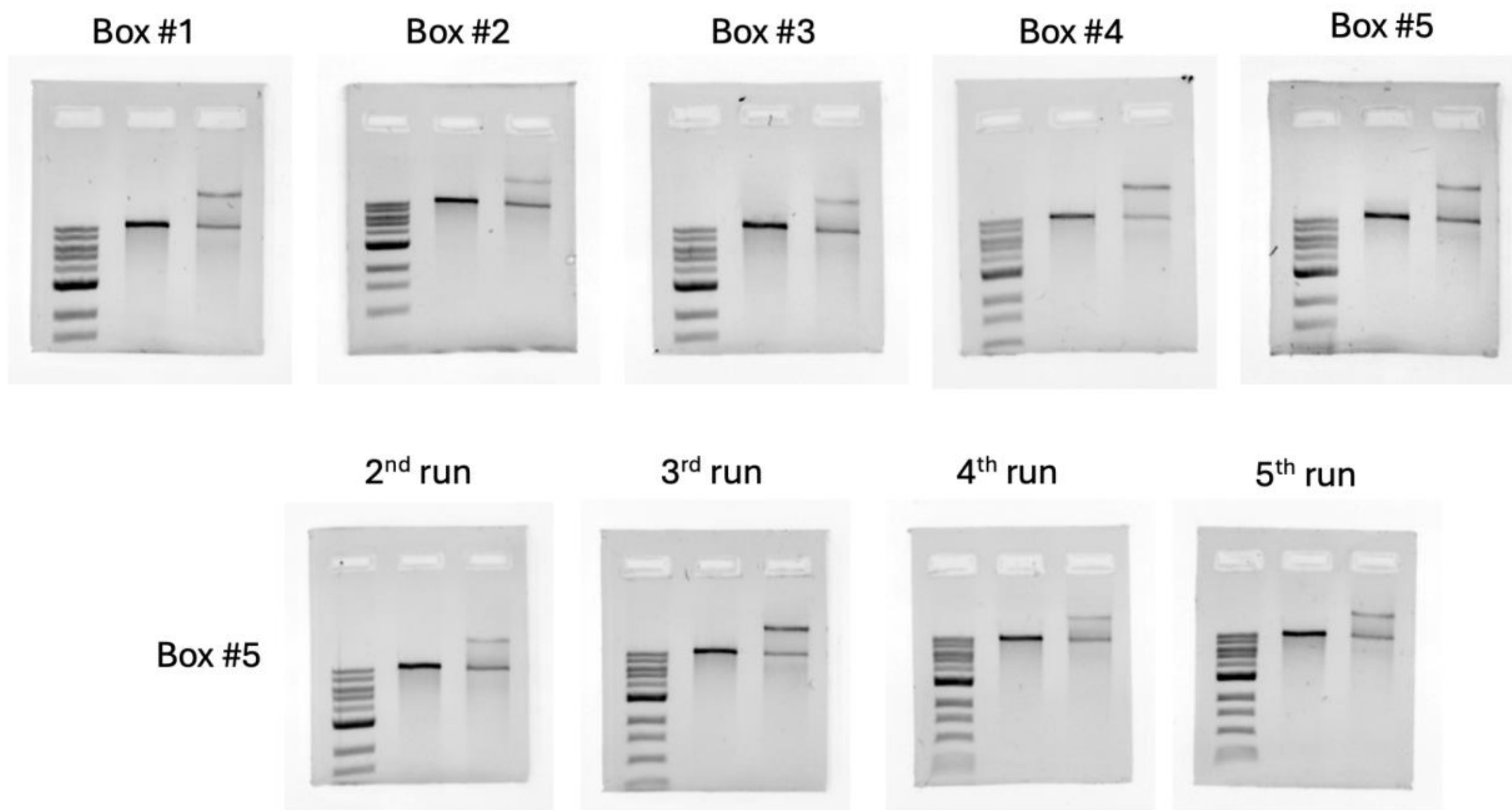

Figure S2. Repeatability of mini gels. Five separate gel boxes were printed and run for the first time, and box #5 was run for a total of 5 times. Lanes in all the gels from left to right: 1= 1kb DNA ladder, 2= negative control, 3=positive control.

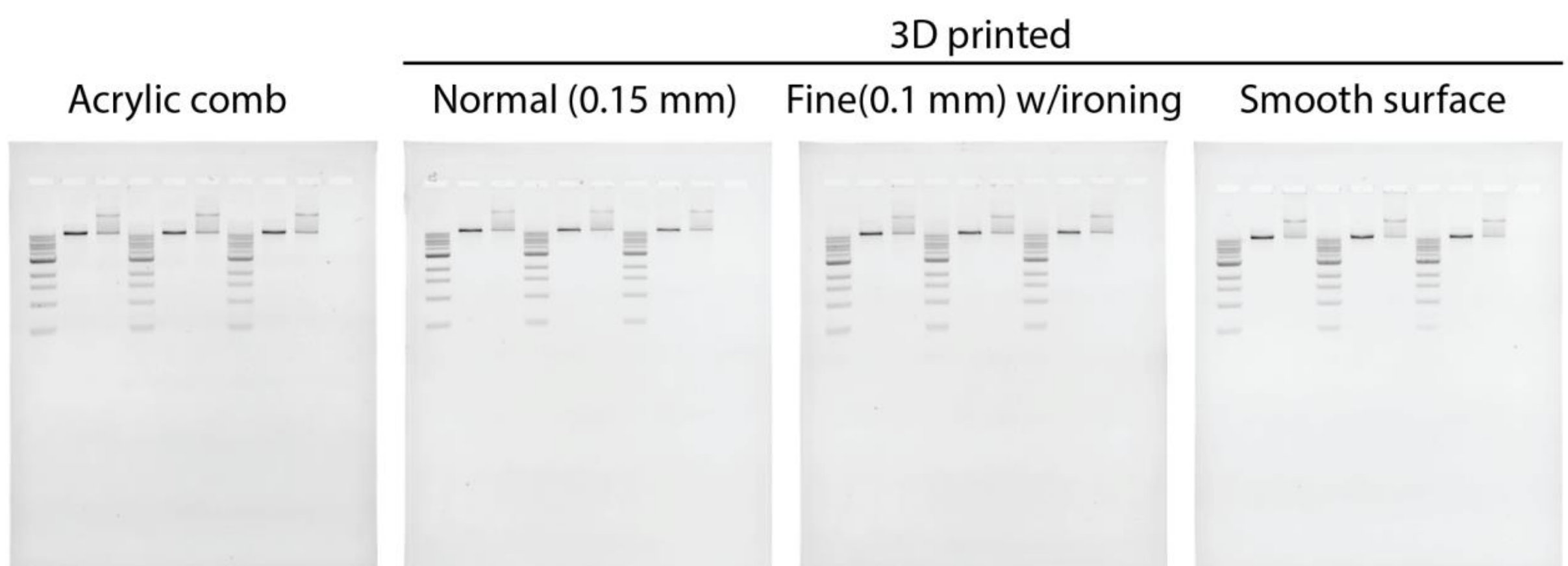

Figure S3. Use of acrylic vs. 3D printed gel combs. Lanes in all the gels in triplicate from left to right: 1= 1kb DNA ladder, 2= negative control, 3=positive control.

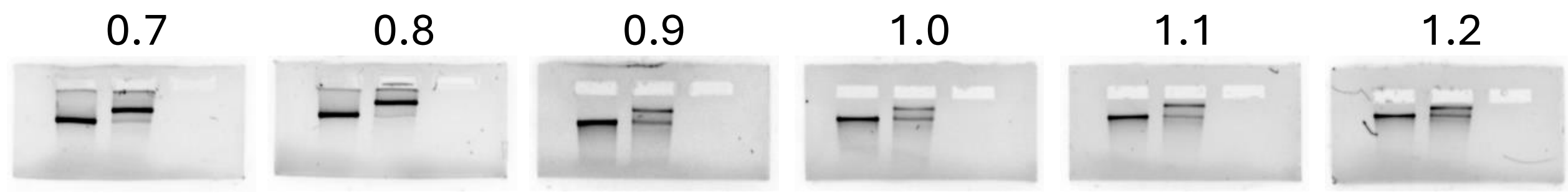

Figure S4. Use of various gel percentages in resolving DNA nanoswitches. Tested different gel concentration between 0.7% to 1.2% w/v with constant running conditions in 15mm gel box. Lanes in all the gels from left to right: negative control, 2=positive control.

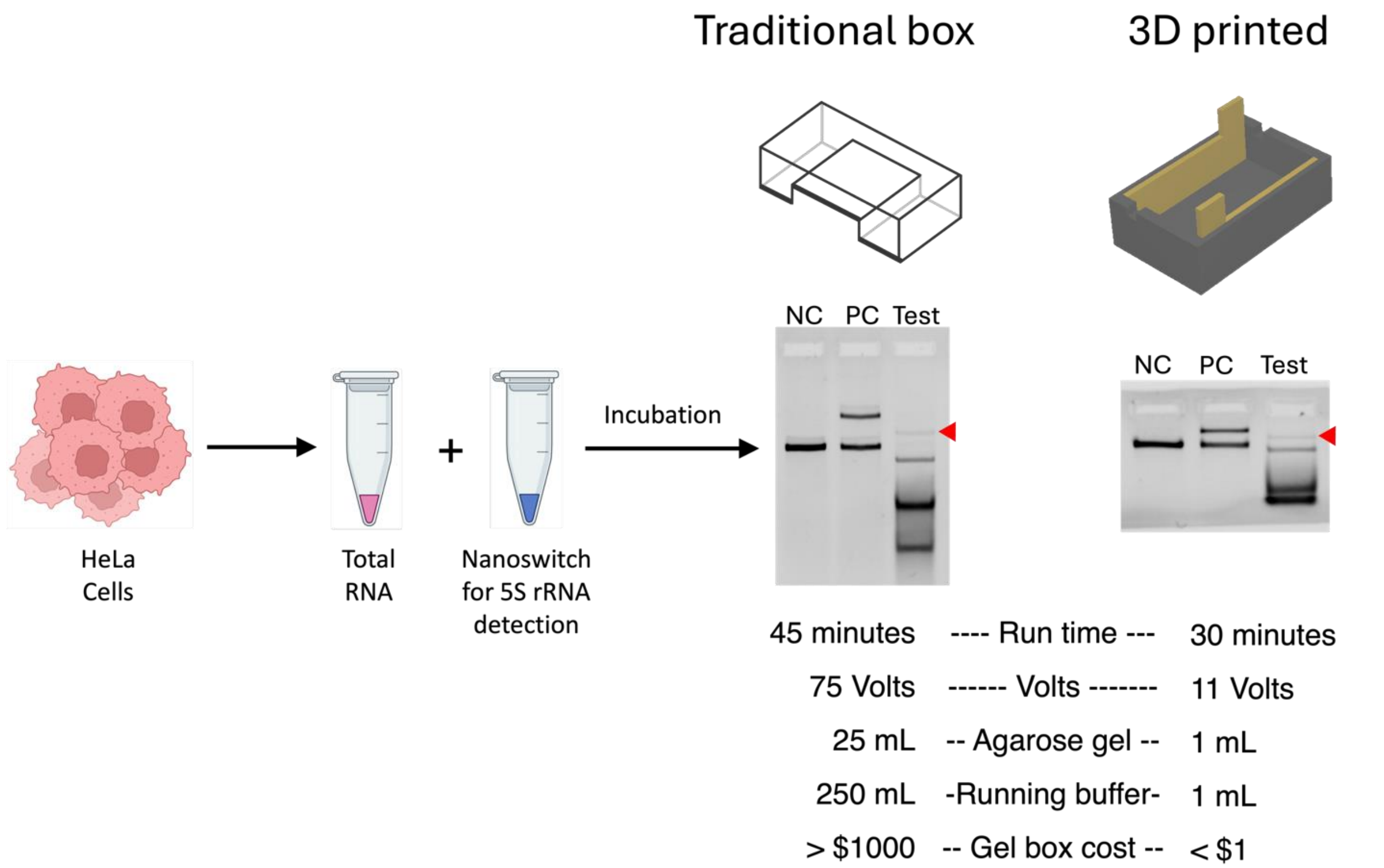

Figure S5. Target detection in a total RNA sample with traditional gel box vs mini-gel box. Detection of 5S rRNA in total RNA sample isolated from HeLa cells. Lane 1= NC (negative control), Lane 2= PC (positive control), Lane 3= test (total RNA sample).
